# Supplementary material for: The impact of work-family conflict on job burnout among community social workers in China
Source: PLoS One. 2025 Mar 12;20(3):e0301614. doi: 10.1371/journal.pone.0301614 (PMC11902123; doi:10.1371/journal.pone.0301614)
Supplement: Attachment — Submitted filename: 20230223 Response to Reviewers.docx [file pone.0301614.s001.docx]

Dear Editors,

We greatly appreciate the opportunity to revise our manuscript entitled " The Impact of Work-Family Conflict on Job Burnout among Community Social Workers in China". The thoughtful guidance and insightful advice provided by you and the reviewers have helped us improve the quality of the manuscript. We have made efforts to take action to address all issues by reviewers in the revision. The modifications made to every comment are presented in detail below.

Thank you again for all the insightful guidance and encouraging comments. We hope you and the reviewers will find our revision satisfactory for further consideration.

Best regards,

Authors.

**Reviewer #1**:

Reviewer #1: The paper is well structured and well written besides focusing on a relevant and timely topic and merits publication subject to incorporating changes/suggestions on a few areas that need further improvement. These are as follows.

**[R1-1]** The Introduction section needs to develop a proper rationale and motivation for the study. For example, why not build the rationale of the study in the context of COVID-19?

**[Response]** Thanks for your advice. We have refined the introduction and added the background of COVID-19.

*Research has shown that a significant proportion of community social workers suffered from depression(Maddock, 2023), and severe burnout(Wu, Wei, Li, Pang, & Su, 2022), especially during the COVID-19 pandemic(Henriques et al., 2024; Wu et al., 2022).*

**[R1-2]** Throughout the document and especially in the Introduction Section, the author has made claims that need to be properly backed by citations. For example, we can see phrases in the intro like " Many researchers have explored....", "Much has been explored by researchers", and " Some Scholars have realized that...." are a few to mention here. All these phrases need to be backed by relevant citations.

**[Response]** Appreciate your suggestion. Per your suggestions, we have added relevant literature in the past five years and rewritten the expression such as many researchers throughout the document. For example, the rewriting in the introduction section is as follows:

*Community social workers play an essential role in community development(Itzhaki-Braun, 2022). With the development of urbanization, Chinese communities are increasingly reliant on community social workers to meet the needs of residents, and the number of community social workers is growing (H. Jiang, Wang, Chui, & Xu, 2017). In China, the saying "a thousand lines above, a needle below" is often used to describe the busyness of community social workers. Line refers to the various tasks that the community needs to face, needle refers to the community social worker. Due to the complexity and variety of their job, community social workers often need to work overtime. Research has shown that a significant proportion of community social workers suffered from depression(Maddock, 2023), and severe burnout(Wu, Wei, Li, Pang, & Su, 2022), especially during the COVID-19 pandemic(Henriques et al., 2024; Wu et al., 2022)**. Burnout leads to high turnover rates in community social workers, which in turn results in the decline of service quality(H. Jiang et al., 2017). Research has shown that socio-demographic factors，role stress(Tang & Li, 2021), prosocial personality(Toledano & Ruiz-Olivares, 2023), job demands(Tu, Luo, Sitar, & Huang, 2022) and work overtime(S. Jiang, Jiang, & Cheng, 2023) influence the burnout of community social workers.*

**[R1-3]** It seems like some of the author guidelines for formatting have been ignored/not followed in some places. For example, we can see spaces before headings at some places and even the way Citations are done in text in the literature section are not in the standard format.

**[Response]** Thank you for bringing this issue to our attention. We have modified the format according to the journal’s requirement. Citations are processed by Endnote based on the APA format.

**[R1-4]** Most of the literature cited in the text of the document is outdated. More relevant and fresh studies need to be cited. Specially in the Literature Section. Moreover, reference to studies conducted in the Chinese context are highly missing and must be included.

**[Response]** Thanks for pointing this out. We have added more relevant and fresh studies. Meanwhile, we have cited the reference in the Chinese context.

First, more relevant, and novel studies are as follows (only list part of them):

*Bande, B., Jaramillo, F., Fernández-Ferrín, P., & Varela, J. A. (2019). Salesperson coping with work-family conflict: The joint effects of ingratiation and self-promotion. Journal of Business Research, 95, 143-155.*

*Chen, L., & Du, Y. A. (2023). The impact of role overload on job crafting from the perspective of construal level theory. Personnel Review, 52(5), 1612-1629.*

*Chen, P., Xu, Y., Sparrow, P., & Cooper, C. (2023). Compulsory citizenship behaviour and work-family conflict: A moderated mediation model. Current Psychology, 42(8), 6641-6652.*

*Clark, M. A., Early, R. J., Baltes, B. B., & Krenn, D. (2019). Work-Family Behavioral Role Conflict: Scale Development and Validation. Journal of Business and Psychology, 34(1), 39-53.*

Second, Chinese research are as follows:

*Chen, P., Xu, Y., Sparrow, P., & Cooper, C. (2023). Compulsory citizenship behaviour and work-family conflict: A moderated mediation model. Current Psychology, 42(8), 6641-6652.*

*Huo, M.-L., & Jiang, Z. (2023). Work-role overload, work–life conflict, and perceived career plateau: The moderating role of emotional stability. Human Resource Management, 62(6), 867-882.*

*Jia, C. X., & Li, J. C. M. (2022). Work-Family Conflict, Burnout, and Turnover Intention among Chinese Social Workers: The Moderating Role of Work Support. Journal of Social Service Research, 48(1), 12-27*

*Jiang, H., Wang, Y., Chui, E., & Xu, Y. (2017). Professional identity and turnover intentions of social workers in Beijing, China: The roles of job satisfaction and agency type. International Social Work, 62(1), 146-160.*

*Jiang, S., Jiang, C. X., & Cheng, Y. H. (2023). Working Overtime in Social Work Settings: Associations with Burnout, Person-organization Value Congruence and Turnover Intentions among Chinese Social Workers. Human Service Organizations Management Leadership & Governance, 47(1), 28-41.*

**[R1-5]** In Section 2.5 i.e. Job Demand-Resource Model, the phrase "...formed by two kind of factors and the health....." is too long and also seems incomplete and hence conveys no meaning.

**[Response]** We see your concerns. In response to the question, we have revised the description of Job Demand-Resource model.

*The job demands-resources model (JD-R Model) is a representative theory explaining Job burnout and its process and mechanism(Demerouti, Bakker, Nachreiner, & Schaufeli, 2001). JD-R argues that job characteristics can be divided into demand and resource factors. The former refers to the factors that cause work stress, the latter refers to the factors that alleviate work stress (job resources). Job resources play a positive role in relieving stress, and job resources* *play a buffering role against job demands and burnout. JD-R Model points out that there are two paths of loss and gain for employees in work, demand leads to loss, resource leads to gain(Schaufeli & Taris, 2014). JD-R Model also posits that work resources can buffer the loss of high workload employees. JD-R Model deduces that employees in challenging environments can better transform high work resources into high-level job performance(Bakker & Demerouti, 2007).*

**[R1-6]** The Literature Review section needs to be split into two distinct parts i.e., i) Empirical Literature and ii) Theoretical Literature/Underpinnings. Currently the theoretical literature citations and theoretical underpinnings are merged which creates confusion.

**[Response]** Thanks for your advice, per your suggestion, we refined our literature review section. When reviewing past literature, we first elaborated on the connotation of the concept and unpinning theory, and then introduced relevant empirical research.

**[R1-7]** The diagram "Theoretical Framework" seems miss-labelled. It should be re-labelled as "Conceptual Model".

**[Response]** Thanks for pointing this out.	We have re-labelled "Theoretical Framework" as o "Conceptual Model".

**[R1-8]** All the Hypotheses need to be backed/supported with the help of relevant literature. Currently in the document this section does not have any citations which is strange to note.

**[Response]** Thanks for your insightful comments. We have revised the writing style in the hypothesis development section by adding citations. For example.

*According to the definition of work-family conflict, work-family interference refers to the interference of working hours, stress, and negative emotions with daily family life, such as a prolonged lack of family education for children or lack of energy to take care of the elderly due to excessive overtime work. Meanwhile, the family interference dimension mainly refers to family demands, just as excessive family demands can also interfere with work duties. Both interferences promote community social worker to worker overtime, requires them to invest more energy into the work, and puts higher demands on their abilities. Based on the principle of loss and gain in JD-R model, excessive job demands causes in a loss of energy, which in turn leads to job burnout (Demerouti et al., 2001). It will bring individuals experience feelings such as physical fatigue, psychological exhaustion, low achievement (Heras, Rofcanin, Escribano, Kim, & Mayer, 2021). Previous studies have shown that work-family conflict affects the burnout of correctional staff (Lambert et al., 2022), university faculty(Zábrodská et al., 2018), doctors(Y. Wang, Liu, Wang, & Wang, 2012).*

**[R1-9]** The author needs to stick to one type of phrasing the hypotheses. The use of words "in other words" is not appropriate. Moreover, the versions of hypotheses mentioned after "in other words" seem more appropriate and therefore only those should be mentioned.

**[Response]** Thank you very much! We appreciate the constructive comments and make all efforts to write in academic English. Later, if needed, we will seek help from editor agency.

**[R1-10]** The word " Samples" in the heading of Section 4.1 and Table 1 needs to be corrected. It should be "Sample".

**[Response]** Thanks for pointing this out. We have corrected "Samples" as "Sample". Meanwhile, we have made great effort to check other errors.

**[R1-11]** While the Author has mentioned details of the number of respondents and questionnaires returned etc. We still fail to see details of the figures about i) the population ii) No of questionnaires that were distributed/sent/shared iii) The sample selection criteria (eg any formula or procedure followed to arrive at the current number of respondents)

**[Response]** We have seen your concerns. We apologize for the lack of a clear exposition of the part.

First, the information of the survey sample is shown in Table 1 in the appendix.

| Indicators | Index subdivision dimension | Number | proportion/% |
| --- | --- | --- | --- |
| Gender | Male | 113 | 24.5% |
|  | Female | 349 | 75.5% |
| Age | Age 30 and under | 91 | 19.7% |
|  | 31-40 years old | 224 | 48.5% |
|  | 41-50 years old | 121 | 26.2% |
|  | Age 50 and above | 26 | 5.6% |
| Education Degree | Junior college and below | 162 | 35.1% |
|  | Undergraduate course | 292 | 63.2% |
|  | Master’s degree or above | 8 | 1.7% |
| Working years | The following 5 years | 127 | 27.5% |
|  | 5-10 years | 180 | 39.0% |
|  | 11-20years | 131 | 28.4% |
|  | More than 20 years | 24 | 5.1% |
| Social Education Major | True | 34 | 7.4% |
|  | False | 428 | 92.6% |
| Compensation | 3,000 yuan of the following | 25 | 5.4% |
|  | 3,001-4,000 Yuan | 329 | 71.2% |
|  | 4,001-5,000 Yuan | 95 | 20.6% |
|  | More than 5,000 Yuan | 13 | 2.8% |

Second, we have described the data collection procedure:

*The respondents were mainly community social workers in Suzhou, Nantong, Nanjing and Hangzhou City. Due to the constraints of time, funds, and epidemic prevention and control, the survey was conducted by an online questionnaire. 500 questionnaires were distributed, including the respondents' informed consent, which was approved by UCHRP. 474 questionnaires were collected from September 15 to December 15 in 2021. 462 valid questionnaires were collected after excluding questionnaires with too short response time, vacant questions, and questionnaires with the same options for all items. As can be seen from Table 1, most of the respondents have college and less than a college bachelor's degree. The educational characteristic of the sample is consistent with the proportion of bachelor's degrees in the social worker workforce which is about 65%. The surveyors working between 5 and 10 years occupy the proportion of 40% which almost equals the statistical data of 2021. The proportion of women is much higher than that of men, with the ratio of the two being about 1:3, which is consistent with the ratio of occupational characteristics in national statistics, and thus the sample is representative.*

**[R1-12]** A very striking remark is that the Table 1 on Characteristics of the Sample shows that about 75.5% of the sample consists of Female respondents. The author has even also commented on it. However, it seems that this thing is making the sample highly biased towards females only. The Author needs to build justification of this bias or present a proper rationale behind focusing more on females.

**[Response]** Thanks for pointing this out. In China, most community social workers are female. According to the statistics released by Suzhou city, The male to female ratio among community workers in Suzhou is about 1:3, and the sample situation is roughly similar to the statistical data, making the sample representative.

**[R1-13]** Finally, while the author has followed a very rigorous statistical procedure and has derived several findings, a comparison with similar/dissimilar findings in previous literature with citations needs to be incorporated to improve the quality of the discussion on findings.

**[Response]** We sincerely appreciate your suggestions. In the findings section, we have added the comparison.

*First. Work-family conflict affects emotional exhaustion, depersonalization, and personal fulfillment, respectively. This result is consistent with the idea of (Allen, French, Dumani, & Shockley)‘. They argued that when work-family conflict is high, individuals can’t shoulder the job and family responsibilities simultaneously, they will display negative emotions such as anxiety (Allen et al.; Y. Wang et al., 2012). The findings again confirms that family-work conflict as a destructive work demand negatively affects the health of community workers.*

*Second. Role overload mediates the relationship between work-family conflict and burnout, which supports our initial hypothesis. The conclusion is identical to prior researchers’ arguments (Ahmad, 2010; Montani & Dagenais-Desmarais, 2018). They pointed out that role overload leads to an enhanced experience of emotional exhaustion. Therefore, when community social workers are overwhelmed by family and work, role overload can lead to burnout as they do not have the time to engage in additional community work, or even social work, which can lead to burnout.*

*Third. Research has found that cognitive processing moderates role overload and burnout. However, we found job crafting does not moderate the relationship between work-family conflict and job burnout, only partially moderated the relationship between role overload and job burnout. The reason for the inconsistent findings may be that previous studies have not subdivided the dimensions of job crafting and have mainly focused on relational and task crafting(Rastogi & Chaudhary, 2018).*

Thank you again for all your insightful feedback and constructive comments. We are hopeful that you will find this revision satisfactory for further consideration.

**Reviewer #2:**

Overall, it was a good effort.

Thank you very much! We hope you and the reviewers will find our revision satisfactory for further consideration.

**[R2-1]** Rewrite the abstract as it was not depicting the whole article.

**[Response]** Thanks for your advice, per your suggestion, we refined our abstract.

*In China, for community social workers, work-family conflict has become a common phenomenon that may harm their well-being. Based on the analysis of a survey of community social workers in four cities in China, this study demonstrated that community workers' work-family conflict significantly affects burnout, role overload mediates the relation between work-family conflict and burnout, and cognitive crafting negatively moderates the relation between role overload and burnout. The conclusions validate the job crafting theory and enrich the research on job burnout under the JD-R model. The practical significance of the study is that on the one hand, community and individual workers can effectively alleviate burnout by clarifying their roles. On the other hand, it also reminds managers that they should pay attention to the physical and mental health of social workers to enable them to develop healthily.*

**[R2-2]** Add latest references in introduction section, add few lines to describe the importance of the research, research problem should be backed with contemporary references.

**[Response]** Thanks for pointing this out. We have added more relevant and fresh studies. Meanwhile, we have cited the reference in the Chinese context.

*First, more relevant, and novel studies are as follows (only list part of them):*

*Bande, B., Jaramillo, F., Fernández-Ferrín, P., & Varela, J. A. (2019). Salesperson coping with work-family conflict: The joint effects of ingratiation and self-promotion. Journal of Business Research, 95, 143-155.*

*Chen, L., & Du, Y. A. (2023). The impact of role overload on job crafting from the perspective of construal level theory. Personnel Review, 52(5), 1612-1629.*

*Chen, P., Xu, Y., Sparrow, P., & Cooper, C. (2023). Compulsory citizenship behaviour and work-family conflict: A moderated mediation model. Current Psychology, 42(8), 6641-6652.*

*Clark, M. A., Early, R. J., Baltes, B. B., & Krenn, D. (2019). Work-Family Behavioral Role Conflict: Scale Development and Validation. Journal of Business and Psychology, 34(1), 39-53.*

Second, Chinese research are as follows:

*Chen, P., Xu, Y., Sparrow, P., & Cooper, C. (2023). Compulsory citizenship behaviour and work-family conflict: A moderated mediation model. Current Psychology, 42(8), 6641-6652.*

*Huo, M.-L., & Jiang, Z. (2023). Work-role overload, work–life conflict, and perceived career plateau: The moderating role of emotional stability. Human Resource Management, 62(6), 867-882.*

*Jia, C. X., & Li, J. C. M. (2022). Work-Family Conflict, Burnout, and Turnover Intention among Chinese Social Workers: The Moderating Role of Work Support. Journal of Social Service Research, 48(1), 12-27*

*Jiang, H., Wang, Y., Chui, E., & Xu, Y. (2017). Professional identity and turnover intentions of social workers in Beijing, China: The roles of job satisfaction and agency type. International Social Work, 62(1), 146-160.*

*Jiang, S., Jiang, C. X., & Cheng, Y. H. (2023). Working Overtime in Social Work Settings: Associations with Burnout, Person-organization Value Congruence and Turnover Intentions among Chinese Social Workers. Human Service Organizations Management Leadership & Governance, 47(1), 28-41.*

**[R2-3]** The researchers may add a paragraph in methodology section to describe the data collection methods, sampling technique and data analysis method,

**[Response]** Thanks for pointing this out. We have added it.

*4. Methodology*

*4.1. Sample and Data Collection*

*The respondents were mainly community social workers in Suzhou, Nantong, Nanjing and Hangzhou City. Due to the constraints of time, funds, and epidemic prevention and control, the survey was conducted by an online questionnaire. 500 questionnaires were distributed, including the respondents' informed consent, which was approved by UCHRP. 474 questionnaires were collected from September 15 to December 15 in 2021. 462 valid questionnaires were collected after excluding questionnaires with too short response time, vacant questions, and questionnaires with the same options for all items. As can be seen from Table 1, most of the respondents have college and less than a college bachelor's degree. The educational characteristic of the sample is consistent with the proportion of bachelor's degrees in the social worker workforce which is about 65%. The surveyors working between 5 and 10 years occupy the proportion of 40% which almost equals the statistical data of 2021. The proportion of women is much higher than that of men, with the ratio of the two being about 1:3, which is consistent with the ratio of occupational characteristics in national statistics, and thus the sample is representative. The sample profile is shown in Table 1.*

*--------------Insert Table 1 about here ---------------------*

*4.2. Measures*

*The measures were adapted from well-established items. All measures were assessed with a 5-point Likert scale; one (1) represents strongly disagree, and five (5) represents strongly agree. The items of work-family conflict, which were derived (Grandey, Cordeiro, & Crouter, 2005), consists of 2 sub-scales with 11 items. The scales of role overload were derived from (Thiagarajan, Chakrabarty, & Taylor, 2006) with 5 items. Cognitive crafting was measured using 6 items from (Slemp & Vella-Brodrick, 2013). The scale of job burnout was adopted from (Maslach, Jackson, & Leiter, 1997), which consists of 16 items.*

*4.3. Statistical Methods*

*This data analysis used R-studio software. Firstly, descriptive statistical analysis was conducted to calculate the mean and standard deviation of each variable. Secondly, confirmatory factor analysis was conducted to validate the reliability and validity of the scales. Thirdly, correlation analysis and hierarchical regression were conducted to test the hypothesis.*

**[R2-4]** Tables should be presented in an appropriate way.

**[Response]** We have seen your concerns. We apologize for the lack of a clear exposition of the part.

First, the information of the survey sample is shown in Table 1 in the appendix.

| Indicators | Index subdivision dimension | Number | proportion/% |
| --- | --- | --- | --- |
| Gender | Male | 113 | 24.5% |
|  | Female | 349 | 75.5% |
| Age | Age 30 and under | 91 | 19.7% |
|  | 31-40 years old | 224 | 48.5% |
|  | 41-50 years old | 121 | 26.2% |
|  | Age 50 and above | 26 | 5.6% |
| Education Degree | Junior college and below | 162 | 35.1% |
|  | Undergraduate course | 292 | 63.2% |
|  | Master’s degree or above | 8 | 1.7% |
| Working years | The following 5 years | 127 | 27.5% |
|  | 5-10 years | 180 | 39.0% |
|  | 11-20years | 131 | 28.4% |
|  | More than 20 years | 24 | 5.1% |
| Social Education Major | True | 34 | 7.4% |
|  | False | 428 | 92.6% |
| Compensation | 3,000 yuan of the following | 25 | 5.4% |
|  | 3,001-4,000 Yuan | 329 | 71.2% |
|  | 4,001-5,000 Yuan | 95 | 20.6% |
|  | More than 5,000 Yuan | 13 | 2.8% |

**[R2-5]** Researcher should add last five years references in all chapters.

**[Response]** Thanks for pointing this out. We have added more relevant and fresh studies.

*more relevant, and novel studies are as follows (only list part of them):*

*Bande, B., Jaramillo, F., Fernández-Ferrín, P., & Varela, J. A. (2019). Salesperson coping with work-family conflict: The joint effects of ingratiation and self-promotion. Journal of Business Research, 95, 143-155.*

*Chen, L., & Du, Y. A. (2023). The impact of role overload on job crafting from the perspective of construal level theory. Personnel Review, 52(5), 1612-1629.*

*Chen, P., Xu, Y., Sparrow, P., & Cooper, C. (2023). Compulsory citizenship behaviour and work-family conflict: A moderated mediation model. Current Psychology, 42(8), 6641-6652.*

*Clark, M. A., Early, R. J., Baltes, B. B., & Krenn, D. (2019). Work-Family Behavioral Role Conflict: Scale Development and Validation. Journal of Business and Psychology, 34(1), 39-53.*

Thank you again for all your insightful feedback and constructive comments. We are hopeful that you will find this revision satisfactory for further consideration.
